# Ventricular stimulation in patients with myotonic dystrophy type 1 may not predict future ventricular arrhythmias

**DOI:** 10.1016/j.hroo.2024.08.001

**Published:** 2024-08-09

**Authors:** Lukasz Cerbin, Amneet Sandhu, Michael Rosenberg, Christopher Barrett, Rafay Sabzwari, Lohit Garg, Alexis Tumolo, Wendy Tzou, Paul Varosy, Johannes Von Alvensleben, Matthew Zipse, Ryan Aleong

**Affiliations:** 1 Division of Cardiology, University of Colorado Anschutz Medical Campus, Aurora, Colorado; 2 Division of Cardiology, Rocky Mountain Regional VA Medical Center, Aurora, Colorado; 3 Division of Cardiology, Denver Health Medical Center, Denver, Colorado; 4 Division of Cardiology, Children’s Hospital Colorado, Aurora, Colorado

**Keywords:** Myotonic dystrophy, Electrophysiology study, Ventricular arrhythmias, Pacemaker, Implantable cardioverter-defibrillator

## Abstract

**Background:**

Myotonic dystrophy type 1 (DM1) is associated with progressive conduction disease. Furthermore, DM1 patients are at risk ventricular arrhythmias (VAs), although prediction remains difficult. The 2022 Heart Rhythm Expert Consensus Statement gives a IIb recommendation to the use of electrophysiology study (EPS) to risk-stratify patients for VAs. The utility of EPS in predicting the development of VAs, however, has not been explored in this patient population.

**Objective:**

The study sought to examine the natural history of DM1 patients with positive and negative ventricular stimulation (v-stim) during EPS.

**Methods:**

Patients with a history of DM1 undergoing EPS with associated v-stim from 2008 to present were retrospectively identified.

**Results:**

From 2008 to 2022, 26 consecutive DM1 patients presented for EPS with v-stim. Four v-stim protocols were positive for sustained or hemodynamically significant ventricular tachycardia (VT), one of which was induced with 600 doubles, the others with triple extrastimuli. A total of 22 of 26 subjects received a device implant, with 18 receiving permanent pacemakers and 4 implantable cardioverter-defibrillators. All 4 of the patients with positive v-stims underwent ICD implantation. After a mean of 5.7 years of follow-up, 7 patients had sustained VT, 6 of whom had negative v-stims. Of the 4 patients with positive v-stims, only 1 developed sustained VT in follow-up. Other than baseline QT interval at time of EPS, no baseline characteristics were significantly different between patients with and without subsequent VT.

**Conclusion:**

In this single center, v-stim in DM1 patients did not predict clinical VAs, as a vast majority of DM1 patients who developed VAs had negative v-stims.

**Key Findings**

▪ The 2022 Heart Rhythm Expert Consensus Statement gives a IIb recommendation to the use of electrophysiology study (EPS) to risk stratify patients for future ventricular arrhythmias. The utility of EPS in predicting the development of ventricular arrhythmias has not been explored in this patient population.
▪ In this retrospective study an electrophysiology study with ventricular stimulation was not predictive of future ventricular arrhythmias.
▪ In myotonic dystrophy patients type 1 if a dual chamber pacemaker is being placed, placement of a dual chamber ICD should be considered if it is concurrent with the patient’s goals of care.

## Introduction

Myotonic dystrophy (DM), or dystrophia myotonica, is the second most common form of muscular dystrophy, a group of diseases that affect muscle development and function.^1^, ^2^, ^3^ Cardiac abnormalities have been described in both DM type 1 (DM1) and DM2, but the frequency and severity of cardiac issues is higher in DM1; therefore, this investigation focused exclusively on DM1 patients.^4^^,^^5^ Issues related to cardiac conduction in DM1 are well described and arise due to fibrosis in the cardiac conduction system as shown previously by autopsy.^6^^,^^7^ Corroborated by the 2018 American College of Cardiology/American Heart Association/Heart Rhythm Society guidelines for management of patients with bradycardia and conduction system abnormalities, progressive atrioventricular conduction disease represents a common cause for sudden death and an indication for pacemaker implant.^8^^,^^9^ Ventricular arrhythmias (VAs) have also been implicated as a cause of sudden death in a substantial portion of DM1 patients.^10^, ^11^, ^12^ However, despite ongoing work,^11^ risk stratification for patients who would benefit from implantable cardioverter-defibrillator (ICD) placement is less well established. The presence of conduction disease (prolonged PR and QRS intervals) on electrocardiogram, as well as the presence of atrial arrhythmias, have been previously shown to increase the risk of VAs.^5^^,^^9^

Electrophysiology study (EPS), primarily to determine the severity and level of conduction system abnormalities, has been used for risk stratification of DM1 patients to evaluate the need for permanent pacemaker therapy.^13^^,^^14^ Despite the fact that ventricular stimulation (v-stim) has been utilized as part of an EPS in prior work and is endorsed in the 2022 Heart Rhythm Society Expert Consensus Statement, the prognostic value of EPS to predict future VAs remains unknown.^5^^,^^15^ To address this gap in knowledge, we sought to investigate whether inclusion of v-stim as part of an EPS in DM1 patients is predictive of future VAs and if there is a potential benefit from ICD implantation. In this single-center retrospective study, the results of the EPS that included v-stim for a cohort of DM1 patients are reported as well as subsequent follow-up for VAs and sudden death.

## Methods

In this analysis, we report procedural and follow-up data from all DM1 patients from 2008 to 2019 who were referred to the University of Colorado Electrophysiology Center and underwent EPS with v-stim to risk-stratify patients for pacemaker or ICD implantation. All patients in our cohort had DM1, which was verified by genetic testing and electromyography performed by the neuromuscular section at our associated neurology department. All DM1 patients had evidence of conduction disease on electrocardiogram with a PR interval >200 ms and/or a QRS interval >120 ms at baseline. None of the patients had evidence of prior documented VAs as detected on nonimplantable monitors. The details of the EPS protocol, inclusive of v-stim, are noted subsequently. The primary cohorts of interest included (1) EPS positive (those in whom EPS resulted in induction of a sustained VA or a VA that resulted in hemodynamic compromise requiring pace termination or cardioversion) and (2) EPS negative (those without induction of VAs or with nonspecific findings or nonsustained VAs during EPS). Patients were evaluated at least annually at our center during follow-up. Follow-up visits included clinical assessment, 12-lead electrocardiograms, and device interrogations if relevant. Remote device transmissions were sent and reviewed quarterly, if possible. Routine use of cardiac magnetic resonance imaging (MRI) was not part of regular follow-up during this timeframe. The study complied with the Declaration of Helsinki and was approved by the University of Colorado Institutional Review Board.

### Electrophysiology study

All patients with DM1 who had an EPS between 2008 and 2019 are reported in this analysis. Standard evaluation of the conduction system was undertaken with measurement of the AH interval, HV interval, and AV Wenckebach cycle length. V-stim was performed from 2 sites, most commonly the right ventricular apex and the right ventricular outflow tract. Utilizing a routine protocol, up to 3 ventricular extrastimuli were delivered with a drive cycle length of 600 ms and 400 ms. V-stim did include long short ventricular extrastimuli to try to induce bundle branch re-entry ventricular tachycardia (VT) given that this arrhythmia is commonly observed in DM1 patients. V-stim was deemed to be positive when sustained monomorphic VT was present after delivery of up to 3 extrastimuli or ventricular fibrillation or polymorphic VT was present after 2 extrastimuli. A sustained VA was defined as those that lasted for at least 30 seconds. V-stim was also considered positive if a ventricular arrythmia was elicited that required pace termination or cardioversion due to hemodynamic compromise. Use of an isoproterenol infusion during the EPS was at the operator’s discretion and not mandated as part of the EPS protocol. Placement of a permanent pacemaker or ICD was determined by the attending electrophysiologist in conjunction with a patient-centered shared decision-making strategy.

### Statistics

The primary endpoint of this study was recurrent sustained VAs at long term follow-up, which consisted of either sustained VT or ventricular fibrillation. In those who received ICDs, a sustained VA was defined as one that required appropriate ICD therapy, including antitachycardia pacing or were monitored in the “untreated” zone. ICD therapies were programmed to minimize unnecessary ICD shocks or antitachycardia therapies and mostly programmed in accordance with the MADIT-RIT (Multicenter Automatic Defibrillator Implantation Trial–Reduce Inappropriate Therapy) trial.^16^ Patient characteristics were compared between those with positive and negative v-stim protocols as well as between those who did and did not meet the first occurrence of sustained VAs, which was defined as the primary endpoint.

Continuous variables are expressed as mean ± SD and discrete variables are expressed as frequency count and percentage. Differences between continuous variables were analyzed using the *t* test and Wilcoxon rank sum test. Differences between discrete variables were evaluated by the chi-square and Fisher exact tests. Stata (version 17; StataCorp) software was used for all analysis. Statistical significance was defined with a 2-sided *t* test with *P* value <.05.

## Results

**Figure 1:**
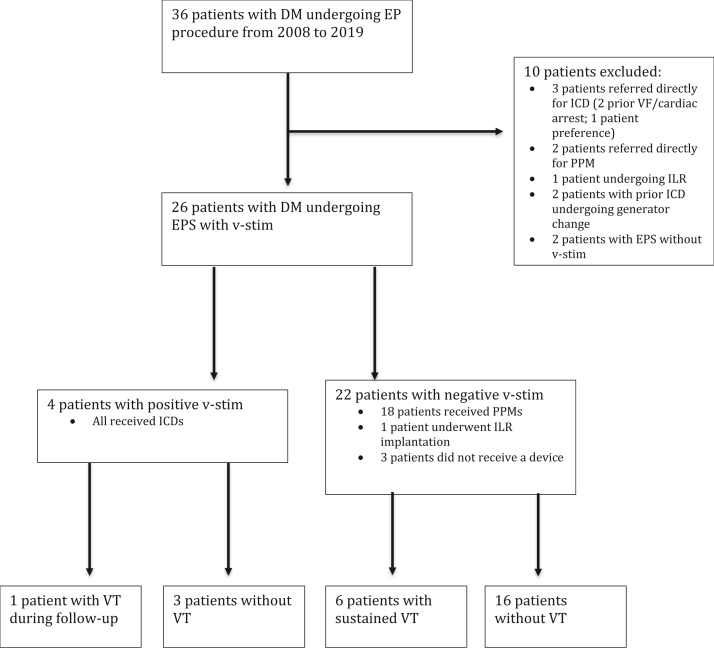
Study flow. DM = myotonic dystrophy; EP = electrophysiology; EPS = electrophysiology study; ICD = implantable cardioverter-defibrillator; ILR = implantable loop recorder; v-stim = ventricular stimulation; PPM = permanent pacemaker; VF = ventricular fibrillation; VT = ventricular tachycardia.

From 2008 to 2019, a total of 81 patients were evaluated in the cardiac electrophysiology clinic to evaluate whether a pacemaker or defibrillator implant was needed. Of those 81 DM patients, 36 DM patients had evidence of conduction disease on their electrocardiogram in clinic with either a PR interval >220 ms or QRS >120 ms, so it was decided to proceed with a cardiac EPS. The demographic and genetic characteristics of the DM1 patients are shown on Table 1. Although the median DMPK CTG repeat length for our cohort of DM1 is reported, it should be noted that the diagnosis of DM1 was made in conjunction with the physical exam performed by the neuromuscular physicians. Of the 36 patients with confirmed DM and conduction disease on EKG who underwent EPS, 26 underwent a concomitant v-stim protocol (Figure 1).

**Table 1:** Baseline patient characteristics (n = 26)

Age, y	51 ± 12.6
Male	17 (65.4)
Hypertension	1 (3.8)
Diabetes mellitus	2 (7.7)
CAD	1 (3.8)
HFrEF	2 (7.7)
LV ejection fraction, %	62 ± 9.4
History of syncope	5 (19.2)
Baseline PR, ms	214 ± 73
Baseline QRS, ms	137 ± 37.2
Baseline QT, ms	442 ± 64.1
AH at EPS, ms	110 ± 58.1
HV at EPS, ms	69.5 ± 24.7
AVWBCL, ms	390 ± 152
Positive v-stim	4 (15.4)
PPM implanted	18 (69.2)
*DMPK* gene CTG Repeat	525 ± 105
ICD implanted	4 (15.4)
Any ventricular tachycardia during follow-up	9 (34.6)
Sustained ventricular tachycardia	7 (26.9)
Death during follow-up	9 (34.6)

Values are mean ± SD or n (%).

AVWBCL = AV Wenckebach cycle length; CAD = coronary artery disease; HFrEF = Heart Failure with reduced Ejection Fraction (LVEF < 40%); EPS = electrophysiology study; ICD = implantable cardioverter-defibrillator; LV = left ventricular; PPM = permanent pacemaker; v-stim = ventricular stimulation.

Among the cohort who underwent v-stim, 67% were male with mean age of 51 ± 12.6 years (Table 1). The average follow-up period was 5.7 ± 3.98 years. The mean left ventricular ejection fraction was 62 ± 9.4%. Five (19%) patients reported a prior history of syncope, and no patients had a history of documented VAs. Baseline conduction intervals, including PR, QRS, AH, and HV, were prolonged (Table 1). A total of 18 (69%) patients underwent pacemaker implantation at the time of EPS and 4 (15%) patients received an ICDs. The remaining 4 patients did not receive a pacemaker or defibrillator since their HV interval was <70 ms and their v-stim was negative.

### Predictive value of v-stim

Baseline characteristics of patients in the EPS-positive and EPS-negative groups are listed in Table 2. Of the total cohort, 4 patients underwent EPS with inducible VAs compared with 22 patients without inducible VAs. Patients with positive v-stim had a lower LVEF on average than those with negative v-stim protocols (51% vs 65%, *P =* .01). Baseline characteristics, including electrophysiologic intervals, were otherwise similar between the groups. None of the patients with negative V-stim protocols received defibrillators, whereas all 4 patients with inducible VAs underwent concomitant ICD implantation.

**Table 2:** Patient characteristics based on v-stim

	Patients with positive v-stim (n = 4)	Patients without negative v-stim (n = 22)	*P* value
Age, y	53.5 ± 16.8	51 ± 12.3	.67
Male	3 (75)	14 (64)	.57
Hypertension	1 (25)	0	.15
Diabetes mellitus	0	2 (9)	.71
CAD	0	1 (5)	.85
HFrEF	1 (25)	1 (5)	.29
LV ejection fraction, %	0.51 ± 0.11	0.65 ± 0.07.1	.01
History of syncope	1 (25)	4 (18)	.60
PR at EPS, ms	233 ± 93.6	218 ± 71.1	.73
QRS at EPS, ms	116 ± 31.4	132 ± 38.3	.43
QT at EPS, ms	405 ± 55.1	436 ± 65.9	.39
AH at EPS, ms	126 ± 71.9	128 ± 57.2	.94
HV at EPS, ms	83.8 ± 22.2	72.2 ± 25.3	.41
PPM implanted	0 (0)	18 (82)	.00
ICD implanted	4 (100)	0 (0)	.00
Any ventricular tachycardia during follow-up	1 (25)	8 (36)	.57
Sustained ventricular tachycardia	1 (25)	6 (27)	.71
Death during follow-up	1 (25)	8 (36)	.57

Values are mean ± SD or n (%).

CAD = coronary artery disease; HFrEF = Heart Failure with reduced Ejection Fraction (LVEF < 40%); EPS = electrophysiology study; ICD = implantable cardioverter-defibrillator; LV = left ventricular; PPM = permanent pacemaker; v-stim = ventricular stimulation.

A positive v-stim during EPS was not predictive of future sustained VAs (Table 2). One of the 4 patients with positive v-stim had subsequent sustained VA over an average of 6.1 years of follow-up. In the group with a negative v-stim at EPS, there were 6 patients with sustained VAs or sudden death during follow-up. There was no significant difference in the occurrence of sustained VAs based on the results of v-stim at EPS (*P =* .57).

### Patients with VT

Of the total cohort, 7 patients were noted to have sustained VAs during follow-up, occurring on average 6 ± 3 years following EPS (Table 3). Some of these patients were followed at outside facilities, so the cycle lengths and the duration of the VAs were not available. Of the 7 patients with an event during follow-up, 1 had an ICD and 6 had PPMs. The patient with an ICD had appropriate therapy for a monomorphic VT. Of the patients with pacemakers, 1 patient had sudden cardiac arrest at home due to VF. The other 5 either presented with VT clinically or had VT detected on their pacemakers. Four of these patients underwent device upgrade to an ICD. The remaining patient did not receive an upgrade to an ICD due to progressive DM1 and a discussion about his goals of care. There were no variables on baseline electrocardiography or EPS, including v-stim, that predicted subsequent sustained VA events (Table 4). Regarding electrocardiograms recorded at patient follow-up, there was a trend indicating an increase in PR and QRS intervals in patients with VAs compared with patients who did not have VAs (Table 4). Given these event rates, v-stim had poor sensitivity (0.14) and positive predictive value (0.25) for predicting future development of VT. The specificity and negative predictive values were 0.84 and 0.73, respectively.

**Table 3:** Description of sustained VAs on follow-up

Patient	Result of v-stim	Device in place	Type of VA (VT/VF)	Method of detection	Symptoms	Time from EPS to VA (y)	Outcome
1	Negative	PPM	VT (duration not documented)	PPM	None documented	11.5	Upgrade to CRT-D
2	Positive	ICD	VT (duration not documented)	ICD	ICD shock	6.6	ICD shock; died during admission
3	Negative	PPM	VF	PPM	Cardiac Arrest	4.8	Death
4	Negative	PPM	VT (duration not documented)	PPM	Palpitations	6.4	Medical therapy
5	Negative	CRT-D (upgraded previously for low LVEF)	VT (duration not documented)	ICD	ATP	10.2	ATP; died 2 mo later
6	Negative	PPM	VT (duration not documented)	PPM	None documented	5.0	ICD upgrade
7	Negative	PPM	VT (38 s)	PPM	None	3.5	Medical therapy

ATP = antitachycardia pacing; CRT-D = cardiac resynchronization therapy with defibrillator; EPS = electrophysiology study; ICD = implantable cardioverter-defibrillator; LVEF = left ventricular ejection fraction; PPM = permanent pacemaker; v-stim = ventricular stimulation; VA = ventricular arrhythmia; VF = ventricular fibrillation; VT = ventricular tachycardia.

**Table 4:** Patient characteristics based on development of sustained VT

	Patients with sustained VT (n = 7)	Patients without sustained VT (n = 19)	*P* value
Age, y	50 ± 13.5	51 ± 12.6	.85
Male	6 (86)	11 (58)	.18
Hypertension	1 (14)	0	.09
Diabetes mellitus	0	2 (11)	.37
CAD	0	1 (5)	.44
HFrEF	1 (14)	1 (5)	.54
LV ejection fraction, %	0.63 ± 9	0.63 ± 9.8	.95
History of syncope	0	5 (26)	.13
PR at EPS, ms	212 ± 73.7	224 ± 74.5	.73
QRS at EPS, ms	108 ± 33.1	137 ± 36.3	.07
QT at EPS, ms	384 ± 44.7	444 ± 63.2	.04
AH at EPS, ms	133 ± 53.8	126 ± 61.5	.78
HV at EPS, ms	68 ± 22.2	76 ± 26.1	.49
PR at most recent follow-up, ms	260 ± 103	199.4 ± 64.8	.10
QRS at most recent follow-up, ms	136 ± 37.9	126 ± 44	.33
PR change during follow-up, ms	22.2 ± 64.0	16.3 ± 49.4	.42
QRS change during follow-up, ms	24.8 ± 24.2	9.3 ± 23.0	.12
Positive v-stim	1 (14)	3 (16)	.92
PPM implanted	6 (86)	12 (63)	.74
ICD implanted	1 (14)	3 (16)	.74

Values are mean ± SD or n (%).

CAD = coronary artery disease; HFrEF = Heart Failure with reduced Ejection Fraction (LVEF < 40%); EPS = electrophysiology study; ICD = implantable cardioverter-defibrillator; LV = left ventricular; PPM = permanent pacemaker; v-stim = ventricular stimulation; VT = ventricular tachycardia.

## Discussion

### Summary of results

In patients with DM1, the current study represents a single-center experience with prolonged follow-up on the ability of v-stim to risk stratify for future VAs and aid in the decision of whether to implant a primary prevention ICD. The key findings of this study are (1) a positive v-stim was not predictive of subsequent sustained VAs after an average of follow-up of 6.1 years and (2) a negative v-stim for VAs may not adequately predict freedom from VAs in DM1 patients. Additionally, there were no baseline characteristics that correlated with the development of future VAs. Based on the findings of this analysis, we would propose that a single v-stim study does not have predictive value to predict future VAs and should not solely dictate if an ICD should be implanted.

### Pathophysiology of myotonic dystrophy

DM1 causes progressive weakness in the face, neck, and distal limb muscles and is related to an expansion of an unstable CTG trinucleotide repeat at the 3′ untranslated region of the *DMPK* gene,^17^ while DM2 causes myotonia and muscle wasting and is caused by an expansion of the CCTG repeat of the *CNBP* gene.^16^

Multiple studies have shown that patients with DM1 have fibrosis and fatty infiltration both of the conduction system as well as the ventricular myocardium.^18^ In 1988, Nguyen and colleagues^19^ showed in autopsy studies that cardiac fibrosis was present in the setting of a normal electrocardiogram and echocardiogram. Ventricular fibrosis may increase the risk of VAs including bundle branch re-entry, which was tested for in our EPSs. However, DM1 patients may have ventricular fibrosis in the setting of an EPS in which no VAs could be induced. Conduction disease has been shown to be progressive in DM1, and it has been recommended that DM1 patients have annual electrocardiograms to evaluate for the need for a pacemaker.^20^^,^^21^ It would therefore stand that progressive fibrosis of the conduction system and myocardium would lower the negative predictive value of v-stim during EPS. This idea is supported by the long event time in our study, which suggests that EPS can risk-stratify DM1 patients in the short term but does not provide long-term risk stratification due to underlying disease progression.

One potential solution would be to perform an EPS with v-stim at regular intervals, but for many DM1 patients this may not be feasible due to their peripheral muscle weakness and other socioeconomic limitations. Wahbi and colleagues^14^ previously noted that an HV interval of 70 ms on EPS was associated with an increased risk of worsening conduction delay and consequently there was a benefit to pacemaker placement. In that study, v-stim was also performed, and an ICD was placed if VAs were induced.^14^ Of note, 4 DM1 patients who did not have induced VTs on EPS subsequently had sudden death due to VAs over 6.1 years of follow-up. While it was useful to perform an EPS with v-stim in this study, it is not indicative of future VAs, as these patients develop a worsening myopathy. At our institution, we have stopped routinely performing an EPS with v-stim; if there is an indication for a pacemaker, a dual-chamber ICD is implanted, provided this is concurrent with the patient’s goals of care.

### Current guideline recommendations

The Heart Rhythm Society Expert Consensus Statement from 2022 for treatment of patients with neuromuscular diseases suggests that a dual-chamber ICD is indicated if the patient has inducible VAs on EPS and states that a dual-chamber ICD may be indicated for DM1 patients in whom a pacemaker implantation is already indicated.^21^ This latter recommendation does not have substantial data to support it; therefore, our data lend further support to empirically placing a dual-chamber ICD if a pacemaker is already indicated. The rationale for our clinical approach to DM1 has been that cardiac involvement is progressive and that a negative v-stim may not predict the future risk of VAs. In this clinical context, a patient-centered shared decision is critical.

Considering our findings that v-stim may not have a prognostic value over long-term follow-up, the treating electrophysiologist and the Heart Rhythm Society need to decide how these patients should be followed. Future studies should consider implantation of an implantable cardiac monitor or routine noninvasive cardiac monitoring. In addition, routine cardiac MRI could be considered to assess ventricular fibrosis. However, additional cardiac monitoring and MRI testing for DM1 patients should consider the individual patient’s overall neuromuscular status, their socioeconomic status, and their desire to potentially have an ICD placed.

### Limitations

This retrospective analysis suggests that v-stim is not predictive of future VAs. This study is limited by its retrospective nature and relatively small study size. These results should prompt further prospective studies of v-stim during EPS in DM1 patients. Additionally, routine monitoring following EPS was not employed, so it is possible that future arrhythmic events were missed, although our hope is that this was mitigated by uniform referral patterns to ensure that there was a similar practice pattern for the entire group of patients. Finally, cardiac MRI was not routinely utilized in this time frame, and its utility in risk stratification of patients with DM1 remains unknown.

### Conclusion

This single-center retrospective study suggests that v-stim during an EPS may not be warranted for DM1 patients, as it is not predictive of future VAs. A pragmatic approach, such as implanting a dual-chamber ICD if there are already signs of conduction disease and there are already plans to place a dual-chamber pacemaker, may be appropriate for DM1 patients. Regardless, pacemaker and ICD implantation in patients with DM1 patients should consider their overall goals of care.
